# Practical screening tools for sarcopenia in patients with systemic sclerosis

**DOI:** 10.1371/journal.pone.0245683

**Published:** 2021-01-22

**Authors:** Vanessa Hax, Rafaela Cavalheiro do Espírito Santo, Leonardo Peterson dos Santos, Mirian Farinon, Marianne Schrader de Oliveira, Guilherme Levi Três, Andrese Aline Gasparin, Nicole Pamplona Bueno de Andrade, Markus Bredemeier, Ricardo Machado Xavier, Rafael Mendonça da Silva Chakr

**Affiliations:** 1 Department of Internal Medicine, Universidade Federal do Rio Grande do Sul (UFRGS), Porto Alegre, Rio Grande do Sul, Brazil; 2 Division of Rheumatology, Hospital de Clínicas de Porto Alegre (HCPA), Porto Alegre, Rio Grande do Sul, Brazil; 3 Division of Rheumatology, Hospital Nossa Senhora da Conceição, Grupo Hospitalar Conceição, Porto Alegre, Rio Grande do Sul, Brazil; Universidade Federal de Goias, BRAZIL

## Abstract

**Introduction:**

In view of the method of diagnosing sarcopenia being complex and considered to be difficult to introduce into routine practice, the European Working Group on Sarcopenia in Older People (EWGSOP) recommends the use of the SARC-F questionnaire as a way to introduce assessment and treatment of sarcopenia into clinical practice. Only recently, some studies have turned their attention to the presence of sarcopenia in systemic sclerosis (SSc).There is no data about performance of SARC-F and other screening tests for sarcopenia in this population.

**Objective:**

To compare the accuracy of SARC-F, SARC-CalF, SARC-F+EBM, and Ishii test as screening tools for sarcopenia in patients with SSc.

**Methods:**

Cross-sectional study of 94 patients with SSc assessed by clinical and physical evaluation. Sarcopenia was defined according to the revised 2019 EWGSOP diagnostic criteria (EWGSOP2) with assessments of dual-energy X-ray absorptiometry, handgrip strength, and short physical performance battery (SPPB). As case finding tools, SARC-F, SARC-CalF, SARC-F+EBM and Ishii test were applied, including data on calf circumference, body mass index, limitations in strength, walking ability, rising from a chair, stair climbing, and self reported number of falls in the last year. The screening tests were evaluated through receiver operating characteristic (ROC) curves. Standard measures of diagnostic accuracy were computed using the EWGSOP2 criteria as the gold standard for diagnosis of sarcopenia.

**Results:**

Sarcopenia was identified in 15 (15.9%) patients with SSc by the EWGSOP2 criteria. Area under the ROC curve of SARC-F screening for sarcopenia was 0.588 (95% confidence interval (CI) 0.420–0.756, *p* = 0.283). The results of sensitivity, specificity, positive likelihood ratio (+LR), negative likelihood ratio (-LR) and diagnostic *Odds Ratio* (DOR) with the EWGSOP2 criteria as the gold standard were 40.0% (95% CI, 19.8–64.2), 81.0% (95% CI, 71.0–88.1), 2.11 (95% CI, 0.98–4.55), 0.74 (95% CI, 0.48–1.13) and 2.84 (95% CI, 0.88–9.22), respectively. SARC-CalF and SARC-F+EBM showed better sensitivity (53.3%, 95% CI 30.1–75.2 and 60.0%, 95% CI 35.7–80.2, respectively) and specificity (84.8%, 95% CI 75.3–91.1 and 86.1%, 95% CI 76.8–92.0, respectively) compared with SARC-F. The best sensitivity was obtained with the Ishii test (86.7%, 95% CI 62.1–96.3), at the expense of a small loss of specificity (73.4%, 95% CI 62.7–81.9). Comparing the ROC curves, SARC-F performed worse than SARC-CalF, SARC-F+EBM and Ishii test as a sarcopenia screening tool in this population (AUCs 0.588 vs. 0.718, 0.832, and 0.862, respectively). Direct comparisons between tests revealed differences only between SARC-F and Ishii test for sensitivity (*p* = 0.013) and AUC (*p* = 0.031).

**Conclusion:**

SARC-CalF, SARC-F+EBM, and Ishii test performed better than SARC-F alone as screening tools for sarcopenia in patients with SSc. Considering diagnostic accuracy and feasibility aspects, SARC-F+EBM seems to be the most suitable screening tool to be adopted in routine care of patients with SSc.

## Introduction

Sarcopenia was originally defined as age-related loss of muscle mass [1]. Recently, the European Working Group on Sarcopenia in Older People (EWGSOP) has updated the operational definition of sarcopenia as a progressive and generalized skeletal muscle disorder that is associated with adverse outcomes including physical disability and mortality [2]. In its 2019 revised definition, EWGSOP2 uses low muscle strength as the primary parameter of sarcopenia and the diagnosis is confirmed by the presence of low muscle quantity or quality [2]. In view of the method of diagnosing sarcopenia being still complex and considered difficult to introduce into routine practice, the EWGSOP2 advises the use of the SARC-F questionnaire as a means of finding individuals with probable sarcopenia so as to carry out its assessment and provide treatment in clinical practice [2].

The SARC-F is a symptom score based on 5 self-reported questions concerning strength, ambulation, rising up from a chair, climbing up a set of stairs, and falls [3]. In longitudinal studies, it has been demonstrated to predict the adverse consequences associated with sarcopenia, such as physical disability, hospitalization, and mortality [4]. Despite SARC-F being easy to conduct, inexpensive, and validated in different populations [5–9], its sensitivity is relatively low, as confirmed in a recent meta-analysis [10]. To overcome this limitation, some authors have combined use of the SARC-F with other features in order to optimize the diagnostic properties of this screening tool (SARC-Calf combining calf circumference [7] and SARC-F+EBM adding age and body mass [5]). In the same way, Ishii test was devised so as to estimate the probability of sarcopenia by using a score based on three variables—age, grip strength, and calf circumference [11].

Systemic sclerosis (SSc) is a rare multisystem autoimmune disease characterized by widespread vasculopathy and progressive fibrosis of the skin and other internal organs such as lungs, gastrointestinal tract, and kidneys [12]. As a systemic inflammatory condition, prominently affecting patients' physical function and nutrition, SSc may be considered a major risk factor for sarcopenia [13–17]. According to different definitions, sarcopenia has been diagnosed in nearly 20% of patients with SSc [14, 15, 18], which is similar to other rheumatic diseases, such as psoriatic arthritis (20%), rheumatoid arthritis (20.8%), and ankylosing spondylitis (22.7%) [19].

Considering that patients with SSc are particularly prone to develop severe clinical complications associated with comorbid sarcopenia, such as physical function decline and death, and that there are several case-finding instruments available not yet validated for SSc, we aimed to compare the sensitivity of SARC-F, SARC-CalF, SARC-F+EBM, and Ishii test as screening tools for sarcopenia in patients with SSc. In addition, we aimed to estimate the other standard measures of diagnostic accuracy and the area under the receiver operating characteristic (ROC) curves as the measurements to describe the accuracy of each screening test.

## Methods

### Patients and study design

A total of 142 consecutive patients with SSc were evaluated between March and December 2019, in a cross-sectional study carried out on a convenience sample of patients diagnosed with SSc followed up at a public university hospital. For an expected prevalence of sarcopenia of 20% in a sample of 94 patients with SSc, we could estimate a power greater than 80% to find a sensitivity ranging from 50% to 85% [8, 11, 20]. Additionally, a *post hoc* calculation retrieved a power of 99% for sensitivity and 54% for specificity. A study flowchart is presented in S1 Fig.

All patients were Brazilian and the vast majority inhabitants of the urban area of Porto Alegre, RS. A standardized and comprehensive research questionnaire was applied to each participant by the same researcher (VH). Disease duration was defined as time from the first non-Raynaud's symptom. Disease subtype was classified as follows: diffuse cutaneous SSc (involving trunk and acral skin), limited cutaneous SSc (restricted to extremities and/or face), or sine scleroderma [12]. The severity of skin disease was evaluated by using the modified Rodnan skin score [21]. Patients also completed the SARC-F questionnaire and data about calf circumference, body mass index, and handgrip strength were collected. Inclusion criterion was the fulfillment of either one of the two mostly used classification criteria for SSc: the ACR/EULAR 2013 classification criteria for SSc [22] and the LeRoy/Medsger 2001 classification criteria for early SSc [23]. Out of the 94 participants, 2 were classified as early SSc patients according to LeRoy/Medsger criteria and 92 were classified as SSc patients according to ACR/EULAR 2013 criteria. Exclusion criteria were: (1) the presence of any overlapping systemic autoimmune disease, (2) severe renal disease, defined as a glomerular filtration rate less than 30ml/min/1.73m^2^, (3) any liver disease, defined as an elevation of aspartate aminotransferase or alanine aminotransferase above three times the upper limit of normal, (4) any chronic infection (e.g., hepatitis C virus, hepatitis B virus, human immunodeficiency virus), (5) severe chronic obstructive pulmonary disease, defined as forced expiratory volume in one second less than 50% of the predicted value, (6) any concomitant malignancy, and (7) any inflammatory myopathy, defined as previous history of myopathy and/or an elevation of creatine phosphokinase CPK or aldolase above 1.5 times the upper limit of normal. This study was conducted according to the principles expressed in the Declaration of Helsinki, all patients signed written informed consent and this research protocol was approved by the institutional Research Ethics Committee of the Hospital de Clínicas de Porto Alegre/Brazil (CAAE 06473019.0.0000.5327).

### Measurements

Body mass was measured with a calibrated digital scale, with participants standing barefoot and wearing light clothes. Body height was measured with a standard fixed stadiometer. Body mass index (BMI) was calculated as weight (kg) per height (m^2^). Maximal calf circumference was measured as the widest circumference of the right calf with the legs relaxed and feet 20 cm apart from each other with an inextensible tape measure, according to the methods previously described [24]. An anthropometric scale with a resolution of 100 g (Filizola S.A. Pesagem e Automação, São Paulo, Brazil), a 1 mm precision stadiometer, and a 1 mm precision measuring tape were used for these measurements.

Handgrip strength was measured using a handheld dynamometer (Jamar Hydraulic Hand Dynamometer, Preston, USA) according to the methods proposed by Roberts *et al* [25]. Patients had to squeeze the device as hard as they could three times in each hand in an alternating manner, and the maximum strength was defined as the highest of the 6 values. Cut-off points to define low strength were <27 kg for men and <16 kg for women according to the EWGSOP2 [2].

The Short Physical Performance Battery (SPPB) was applied to evaluate physical performance [26]. It consists of three separate tests: balance, 4 m gait speed and chair stand test. In the balance test, the patient holds his balance for 10 seconds in three standing positions with eyes open: feet side by side, feet in semi-tandem stance, and feet in tandem stance. Only one attempt was permitted for each stance. In the gait speed test, patients walk a 4-m marked course at their usual walking pace, with the examiner timing their walk with a stopwatch. Two attempts were allowed on this test, with the fastest recorded time being used for the overall score. The chair stand test examines the ability to rise from a sitting to a standing position from an armless chair, with the arms folded across the chest. In the final part of the SPPB, a series of five consecutive chair stands, which should be performed as quickly as possible. The examiner times the patient’s performance with a stopwatch, counting aloud the number of stands completed. A score between 0 and 4 was assigned for each component, reaching a maximum of 12 points. According to the EWGSOP2, SPPB ≤8 defines low physical performance [2].

Body composition was measured by whole-body dual-energy X-ray absorptiometry (DXA) (Lunar Prodigy Primo, GE Medical Systems, UK). Patients were wearing only underwear and were asked to remove all metal accessories and jewelry before measurements, which were taken in the morning. After that, patients were aligned in the center of the densitometer table with the feet positioned together and with the hands positioned with palms flat against the densitometer table (for larger subjects who do not fit within the constraints of the scanning field, hands were placed laterally against the hips). Only one patient had knee prosthesis. Following the EWGOSP2 consensus recommendations to use lean soft tissue assessed by DXA to infer muscle mass quantity [2], the appendicular skeletal muscle mass index (ASMI) was calculated as appendicular skeletal muscle mass (the sum of the muscle mass in both arms and legs) divided by height squared. Considering the cut-off points recommended by the EWGSOP2, men with an ASMI below 7.0 kg/m^2^ and women below 5.5 kg/m^2^ were defined as presenting low muscle quantity.

### Assessment of sarcopenia (EWGSOP2)

Sarcopenia was defined according to the 2019 revised EWGSOP2 criteria [2]. This definition uses low muscle strength (determined by handgrip strength) as the primary parameter of sarcopenia and the diagnosis is confirmed by the presence of low muscle mass (determined by DXA). In the presence of low muscle strength, low muscle quantity and low physical performance (determined by SPPB), sarcopenia is considered severe.

### Sarcopenia screening tools

We used the SARC-F, SARC-CalF, SARC-F+EBM, and Ishii screening test to estimate the presence of sarcopenia. The standard SARC-F is composed of 5 items questioning the strength, assistance in walking, rise from a chair, climb stairs, and self reported number of falls in the last year (each one scored between 0 and 2) [3]. The score ranges from 0–10 and, in the original study, a score equal to or greater than 4 was predictive of sarcopenia and poor outcomes [3]. The original SARC-F questionnaire was already translated to Portuguese and validated as a sarcopenia screening tool in Brazil with the optimal cut-off point equal to or greater than 6 [7]. In the current study, we applied this validated version; however, due to the lack of cut-offs standardization, we performed separate analyses and chose the value with a better performance in our specific sample which was equal to or greater than 4 (S1 File).

The SARC-CalF is composed of 6 items: the standard SARC-F (5 items: strength, walking ability, rising from a chair, stair climbing, and self reported number of falls in the last year) and a sixth additional item (maximal calf circumference) [7]. Calf circumference is measured through scoring: zero representing the absence of low muscle mass (>34 cm for men and >33 cm for women) and 10 for presence (≤34 cm for men and ≤33 cm for women). The score ranges from 0–20. For the SARC-CalF, a total score of ≥11 indicates positive screening for sarcopenia [7].

The SARC-F+EBM is a score that combines SARC-F with data about age and BMI [5]. For age, patients with < 75 years of age scored zero point, whereas ≥ 75 years of age scored 10 points. For BMI, patients not being underweight (>21 kg/m^2^) scored zero point, whereas underweight (≤21 kg/m^2^) patients scored 10 points. The score ranges from 0–30. For the SARC-F+EBM, a total score of ≥12 indicates positive screening for sarcopenia [5].

The Ishii screening test calculates the probability of sarcopenia based on three selected variables: age, grip strength and calf circumference [11]. The formula to calculate the score is as follows: score in men = 0.62 (age– 64)– 3.09 (grip strength– 50)– 4.64 (calf circumference– 42), score in women = 0.80 (age– 64)– 5.09 (grip strength– 34)– 3.28 (calf circumference– 42). Alternatively, this score could be easily obtained from the values of the three variables combined on a simple score chart in each sex. For the Ishii test, a total score of ≥105 in men and ≥120 in women is suggestive of sarcopenia [11].

### Statistical analysis

Statistical analyses were performed by using the Statistical Package for the Social Sciences version 23.0 (SPSS Statistics; IBM, Armonk, NY) and MedCalc Statistical Software version 16.8.4 (MedCalc Software, Ostend, Belgium). Variables with a normal distribution were presented as mean and standard deviation (SD), and non-normal quantitative variables were presented as the median and interquartile range (IQR). Sensitivity, specificity, positive likelihood ratio (+LR), negative likelihood ratio (-LR), positive predictive value (PPV), negative predictive value (NPV), and diagnostic *Odds Ratio* (DOR) were computed by using the EWGSOP2 criteria as the gold standard for diagnosis of sarcopenia. The diagnostic accuracy of the SARC-F, SARC-CalF, SAR-F+EBM, and Ishii screening tests were calculated so as to identify sarcopenia. The overall accuracy of screening tests was evaluated by ROC curves. The area under the ROC curve (AUC) and 95% confidence interval (CI) were calculated for all tests and Youden's J statistics was used to compare the performance of SARC-F with different cut-off values. An AUC greater than 0.9 has high accuracy, whereas 0.7 and 0.9 indicate moderate accuracy, 0.5 and 0.7 low accuracy, and 0.5 a chance result [26]. To compare sensitivity, specificity, +LR, -LR, PPV, NPV, and AUC of the screening tests, we used one-way ANOVA with Tukey HSD ("Honestly Significant Difference") *post-hoc* test to indicate which groups were significantly different from others. There were no missing values of any variable in the entire analytic sample. All statistical tests were 2-sided. A *p* value of less than 0.05 was considered statistically significant.

## Results

Out of 142 patients evaluated initially, 37 were excluded for not meeting inclusion criteria. Of these 105 patients, 11 patients refused to participate, remaining a total of 94 patients diagnosed with SSc (7 men and 87 women). The mean age mean age of the total sample was 60.5±10.3 years (range 33–79 years of age). Table 1 shows the clinical characteristics of patients with SSc stratified by sex.

**Table 1:** Clinical characteristics of patients with SSc, stratified by sex.

Characteristics	Women (n = 87)	Men (n = 7)
Age (years)^a^	60.8 ±10.2	56 (23)
Caucasian	71 (81.6)	6 (85.7)
Smoking status		
Never	48 (55.1)	2 (28.6)
Previous	31 (35.6)	4 (57.1)
Current	8 (9.2)	1 (14.3)
Diffuse skin involvement	17 (20)	3 (42.9)
Rodnan Skin Score^a^	4 (8)	11 (9)
Disease duration^a^	12.8 (12.3)	9.1 (12.6)
Anthropometric measures^a^		
Weight (kg)	64.8 ±11.7	70.6 (6.8)
Height (cm)	158 ±6.2	174 (6)
BMI (kg/m^2^)	25.9 ±4.7	22.3 (6.7)
Calf circumference (cm)	35.8 ±3.7	35.2 (5.5)
Years of formal education		
< 2 years	38 (43.7)	3 (42.9)
2–10 years	19 (21.8)	4 (57.1)
> 10 years	30 (34.5)	0 (0)
Handgrip strength (kg)^a^	18 (13)	26 (9)
SPPB^b^ (points)^a^	10 (2)	10 (1)
Gait speed (m/s)^a^	1.09 ±0.33	1.07 (0.24)
ASMI (kg/m^2^)^a^	6.4 ±0.8	7.6 (1.2)

Data are presented as number (percentage) of patients, except when indicated otherwise.

^a^ Data are presented as mean ±standard deviation or median (interquartile range).

^b^ SPPB: 0–12 points being the score range.

Abbreviations: ASMI: appendicular skeletal muscle mass index; BMI: body mass Index; SPPB: short physical performance battery; SSc: systemic sclerosis.

**Fig 1:**
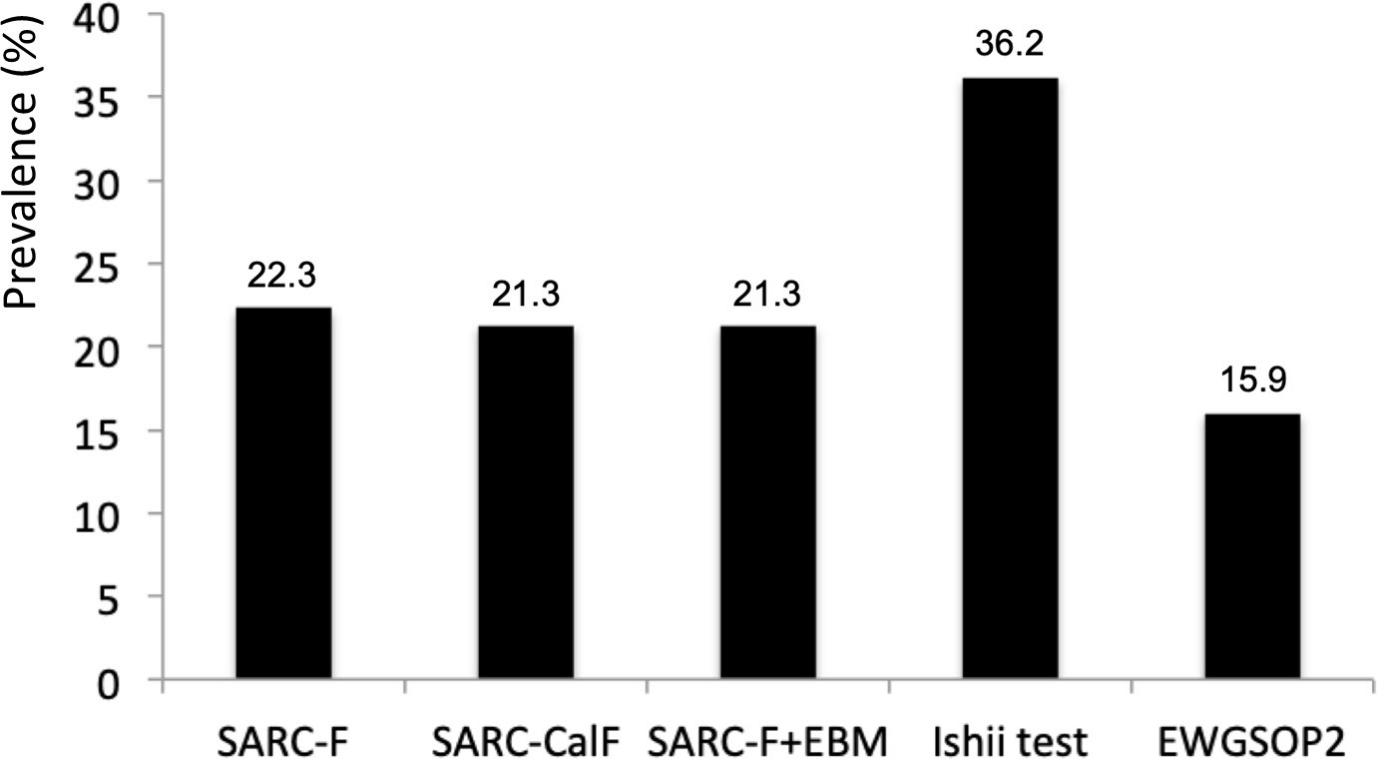
Prevalences of sarcopenia according to each screening tool and diagnostic gold standard (EWGSOP2).

Sarcopenia was identified in 15 patients with SSc (15.9%) by the EWGSOP2 criteria (Fig 1) and severe sarcopenia in 5 patients (5.3%). Average (SD) scores for screening tools were: SARC-F 2.56 (1.84), SARC-CalF 5.12 (4.96), SARC-F+EBM 5.01 (5.12), Ishii test 96.31 (37.75).

**Fig 2:**
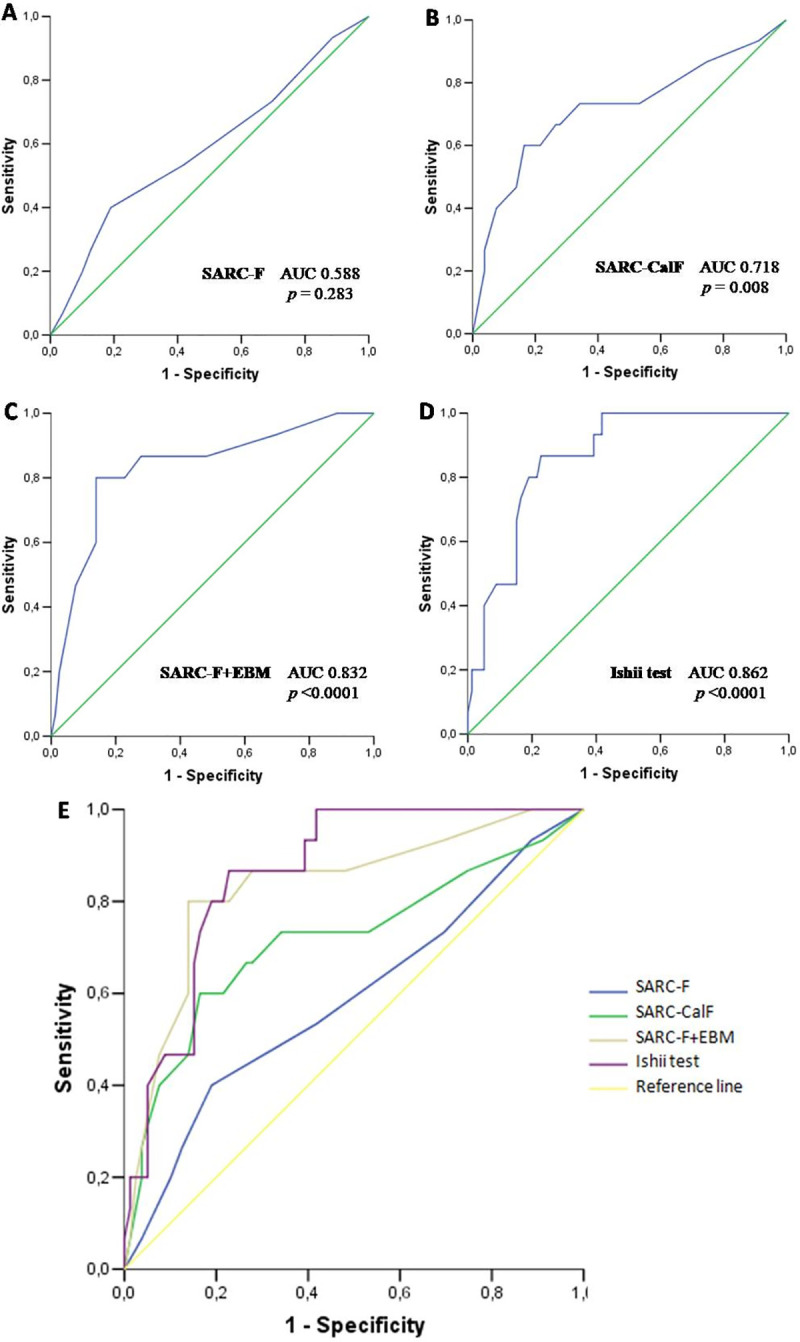
Receiver operating characteristic (ROC) curve analysis of four sarcopenia screening tests in relation to sarcopenia defined according to EWGSOP2 criteria: SARC-F (A), SARC-CalF (B), SARC-F + EBM (C), and Ishii screening test (D). Comparison of ROC curves between the SARC-F, SARC-CalF, SARC-F + EBM, and Ishii’s scores for the sarcopenia screening (E).

Concerning the ability to evaluate sarcopenia, the ROC curves of the four screening tests against the EWGSOP2 definition of sarcopenia are shown in Fig 2.

Table 2 presents the results of sensitivity/specificity analysis and AUC of these tests in the whole study population by using EWGSOP2 diagnostic criteria as the reference standard. The raw data of each tool vs. the gold standard diagnostic results were provided in 2x2 tables as S1 File. Area under the ROC curve of SARC-F screening for sarcopenia was 0.588 (95% CI 0.420–0.756, *p* = 0.283). The SARC-F results of sensitivity, specificity, +LR, -LR, and DOR with the EWGSOP2 criteria as the gold standard were 40.0% [95% CI, 19.8–64.2], 81.0% (95% CI, 71.0–88.1), 2.11 (95% CI, 0.98–4.55), 0.74 (95% CI, 0.48–1.13) and 2.84 (95% CI, 0.88–9.22), respectively. The optimal cut-off point of SARC-F in our sample was ≥4 (Youden index: 0.21), the same cut-off point recommended in the literature [3, 10]. Only 6 (40%) out of the 15 participants with sarcopenia were identified by the SARC-F questionnaire in our population. However, the SARC-F properly identified 4 out of 5 patients who had severe sarcopenia.

**Table 2:** Test characteristics and Receiver Operating Curve (ROC) models for the screening tests for identifying sarcopenia (EWGSOP2).

Screening test	Sensitivity*^¶^	Specificity*	Positive predictive value*	Negative predictive value*	Positive likelihood ratio	Negative likelihood ratio	AUC^¶^
SARC-F	40.0 (19.8–64.2)	81.1 (71.0–88.1)	28.6 (15.6–46.3)	87.7 (82.3–91.6)	2.11 (0.98–4.55)	0.74 (0.48–1.13)	0.588 (0.420–0.756)
SARC-CalF	53.3 (30.1–75.2)	84.8 (75.3–91.1)	40.0 (24.8–57.4)	90.5 (84.7–94.3)	3.51 (1.74–7.09)	0.55 (0.32–0.95)	0.718 (0.553–0.882)
SARC-F+EBM	60.0 (35.7–80.2)	86.1 (76.7–92.0)	45.0 (29.2–61.9)	91.9 (85.8–95.5)	4.31 (2.17–8.56)	0.46 (0.25–0.87)	0.832 (0.713–0.952)
Ishii test	86.7 (62.1–96.3)	73.4 (62.8–81.9)	38.2 (28.9–48.4)	96.7 (88.8–99.1)	3.26 (2.15–4.95)	0.18 (0.05–0.66)	0.862 (0.781–0.944)

* Values are % (95% confidence interval).

^¶^ *p*<0.050 by one-way ANOVA among SARC-F, SARC-CalF, SARC-F+EBM and Ishii test.

Abbreviators: AUC: area under the curve; EWGSOP: European Working Group on Sarcopenia in Older People.

As summarized in Table 2, the magnitude of the sensitivity could vary widely: from 40% for the SARC-F alone to 86.7% for the Ishii screening test. SARC-CalF showed better sensitivity (53.3%, 95% CI 30.1–75.2) and better specificity (84.8%, 95% CI 75.3–91.1) compared with SARC-F. The same occurred with the SARC-F + EBM, that presented better sensitivity (60.0%, 95% CI 35.7–80.2) and also a slightly better specificity (86.1%, 95% CI 76.8–92.0) than SARC-F alone and SARC-CalF. The best sensitivity (86.7%, 95% CI 62.1–96.3) and the best NPV (96.7%, 95% CI 88.8–99.1) were obtained with the screening test of Ishii *et al*, at the expense of a relatively small loss of specificity (73.4%, 95% CI 62.7–81.9). In contrast, the most specific tool was the SARC-F+EBM (86.1%, 95% CI 76.8–92.0), which also presented de highest +LR (4.31, 95% CI 2.17–8.56) and PPV (45%, 95% CI 29.2–61.9).

Comparing the aforementioned ROC curves (Fig 2), SARC-F performed worse than the SARC-CalF, SARC-F+EBM and Ishii test as a sarcopenia screening tool (AUCs 0.588 vs. 0.718, 0.832, and 0.862, respectively).

Additionally, when all tests were evaluated together, there were no differences among screening tests for specificity (*p* = 0.156), PPV (*p* = 0.473), NPV (*p* = 0.077), +LR (*p* = 0.639) and -LR (*p* = 0.098), whereas sensitivity (*p* = 0.020) and AUC (*p* = 0.026) were statistically different. *Post-hoc* direct comparisons between tests revealed differences only between SARC-F and Ishii test for sensitivity (*p* = 0.013) and AUC (*p* = 0.031).

## Discussion

An ideal screening test has to combine a reasonably high sensitivity to find cases in the tested population with a relative high specificity to reduce the number of false positives, avoiding unnecessary and expensive investigations [9, 27]. Aligned with results of previous reports, our study demonstrated that SARC-F presents a poor sensitivity but a high specificity [3–5, 7–9]. Also, in a recent meta-analysis including 7 studies (12,800 subjects), the pooled results of SARC-F sensitivity, specificity, and DOR with the EWGSOP first criteria as the gold standard were 21% (95% CI, 13–31), 90% (95% CI, 83–94), and 2.47 (95% CI, 1.64–3.74), respectively(10). In our sample, 15.9% of patients with SSc had sarcopenia, according to the EWGSOP2 criteria (gold standard), and SARC-F sensitivity, specificity and DOR were 40% (95% CI, 19.8–64.2), 81% (95% CI, 71.0–88.1) and 2.84 (95% CI, 0.88–9.22), respectively. Even though our findings indicate a relatively greater sensitivity, it still lacks clinical utility as 60% of patients with sarcopenia will test negative on SARC-F. The highest sensitivity was from Ishii test, according to which 13.3% of patients with sarcopenia will test negative. A screening tool for sarcopenia presenting high sensitivity is important for prompt identification of patients at risk in clinical practice, allowing to start at the earliest diagnostic confirmation and preventive strategies [9].

On the other hand, the diagnostic accuracy of a screening tool also could be assessed using the AUC value. According to this approach, the observed performance of SARC-F as a screening tool for sarcopenia (AUC 0.588) is, therefore, considered insufficient, suggesting that SARC-F questionnaire is not an adequate tool for sarcopenia screening in patients with SSc. In our study, SARC-CalF, SARC-F+EBM, and Ishii test proved to be superior to SARC-F alone for sarcopenia screening, all of them presenting AUC greater than 0.7.

In practical terms, PPV indicates the probability of having sarcopenia when the test is positive and the NPV, the probability of not having sarcopenia when the test is negative. Generally, previous studies have indicated higher NPV than PPV for sarcopenia screening tools [4–8]. In our study, SARC-F presented the lowest PPV and NPV, and SARC-F+EBM the highest PPV and Ishii test the highest NPV.

In 2016, the (Brazilian) Portuguese-translated version of the SARC-F questionnaire was validated in a population-based study [7]. These authors also proposed to improve its efficacy by associating SARC-F to calf circumference, as an estimate of muscle mass [7]. The SARC-CalF significantly improved SARC-F’s screening performance (AUC 0.736 vs. 0.592, *p* = 0.027), with a substantial increase in sensitivity (SARC-F 33% vs. SARC-CalF 66%) without compromising the remaining parameters [7]. In a recent meta-analysis, including 5 studies (1,127 participants), the pooled results of sensitivity, specificity, and AUC with the EWGSOP first criteria as the gold standard were 58% (95% CI 46–70), 87% (95% CI 84–90), and 0.860 (95% CI 0.83‒0.89), respectively [6]. In our study SARC-CalF also presented a significantly higher sensitivity, specificity and AUC compared to SARC-F alone in patients with SSc (53%, 84% and 0.718, respectively). Adopting a different approach, Kurita *et al* proposed to add “EBM” (“elderly” and “body mass” index information) to SARC-F in order to improve its diagnostic accuracy in patients with musculoskeletal disease [5]. Using the EWGSOP2 criteria as the reference standard, SARC-F+EBM presented higher sensitivity (84.2% vs. 47.4%) and AUC (0.876 vs. 0.558) than SARC-F alone. Thus, the authors suggested that SARC-F+EBM may be a better approach to finding cases of sarcopenia in patients with musculoskeletal disease [5]. In our study, SARC-F+EBM also presented significantly higher sensitivity and AUC than SARC-F in patients with SSc (60% and 0.832, respectively) and also the best specificity, +LR and PPV among the other tests evaluated (86%, 4.31, and 45%, respectively). In the original validation study of SARC-F+EBM, patients were selected after referral for spinal surgery or knee or hip replacement therapy and osteoarthritis was the most common diagnosis [5]. Even though SSc patients may present associated osteoarthritis, in the present study severe functional limitation due to osteoarthritis was not frequent. Considering the specific clinical features of SSc that may contribute to sarcopenia, such as skin thickening and interstitial lung disease, we understand that the best performance of SARC-F+EBM in our study is not predominantly due to similarities with the original study's population.

Aware of these SARC-F’s limitations, EWGSOP2 consensus mentions that clinicians may prefer a more formal case-finding tool to be used in populations where sarcopenia is likely [2, 9], suggesting the Ishii screening test as an option in this setting [11]. Applying this method, the probability of sarcopenia could be easily obtained from a score chart in each sex, combining three variables—age, grip strength, and calf circumference [11]. When the sum of sensitivity and specificity was maximized, sensitivity, specificity, and AUC for sarcopenia were 85%, 88%, and 0.939 for men, and 75%, 92%, and 0.909 for women, respectively. In our sample, the Ishii test also presented the best sensitivity (87%), NPV (96.7%) and–LR (0.182), at the expense of a small decrease in specificity (73%).

An important aspect to be considered is the choice of the cut-off values for sarcopenia definition. According to the EWGSOP2 consensus, reference values were provided to increase harmonization of sarcopenia studies [2]. In a previous regional study, Barbosa-Silva *et al*. used a different cut-off for ASMI, since the value recommended by EWGSOP2 consensus was not able to identify low muscle mass within their sample [28]. In contrast to the study by Barbosa-Silva *et al*, the present study, using EWGSOP2 consensus reference values, identified a prevalence of sarcopenia similar to those reported in previous studies of SSc patients [13–18]. Therefore, instead of using the adapted cut-off values, we chose to report our findings using the reference values recommended by EWGSOP2.

To the best of our knowledge, the present study was the first attempt to evaluate the diagnostic accuracy of the SARC-F questionnaire in a sample of patients diagnosed with SSc. As previously described, our results confirmed the low sensitivity of SARC-F [10] and the better diagnostic accuracy of other tests compared to SARC-F [5–7, 11, 29–34], but in a different population with a high reported prevalence of sarcopenia. Therefore, we understand that the SARC-F+EBM combines the best set of diagnostic properties with the easiest application into clinical practice since it does not depend on the handgrip strength as Ishii test (dynamometers are widely available in research centers, but hardly ever present in doctors’ offices). In the context of personalized medicine, the proper choice of a screening strategy using easily applicable tools could provide relevant diagnostic information about sarcopenia in patients with SSc.

Our study should be interpreted within its limitations. The sample size may not be large enough to detect some differences in accuracy of the screening tests in some subgroups of patients, especially among men (only 7 patients) and non-Caucasian (only 17 patients). Also, a limited sample size could be the reason why there was no difference for most diagnostic measures among the tests, as only sensitivity and AUC were different between SARC-F and Ishii test. In addition, due to our cross-sectional design it was not possible to address the direct impact of a positive screening test in disability, hospitalizations and mortality, as previously shown in other studies [4, 35, 36]. Moreover, our comprehensive exclusion criteria could potentially cause selection bias and limit our findings' external validity. Finally, considering the clinical features of our sample that may interfere on sarcopenia measures, such as skin thickening, joint disease, interstitial lung disease and pulmonary hypertension, we acknowledge the limitations of using previously validated tools on a different population and encourage the development of specific tests for SSc patients.

## Conclusion

In view of sensitivity, PPV, +LR, -LR, DOR and AUC, SARC-CalF, SARC-F+EBM, and Ishii test performed better than SARC-F alone as screening tools for sarcopenia in patients with SSc diagnosed by EWGSOP2 criteria. Only specificity and NPV were greater in SARC-F. Considering diagnostic accuracy and feasibility aspects, SARC-F+EBM seems to be the most suitable screening tool to be adopted in routine care of patients with SSc. These findings need validation in larger samples and different settings, preferably in a longitudinal design to assess the prognostic properties of each screening test.

## Supporting information

S1 File (ODT)

S1 Fig (TIF)
