# Supplementary figures and images for: Practical screening tools for sarcopenia in patients with systemic sclerosis
Source: PLoS One. 2021 Jan 22;16(1):e0245683. doi: 10.1371/journal.pone.0245683 (PMC7822499; doi:10.1371/journal.pone.0245683)

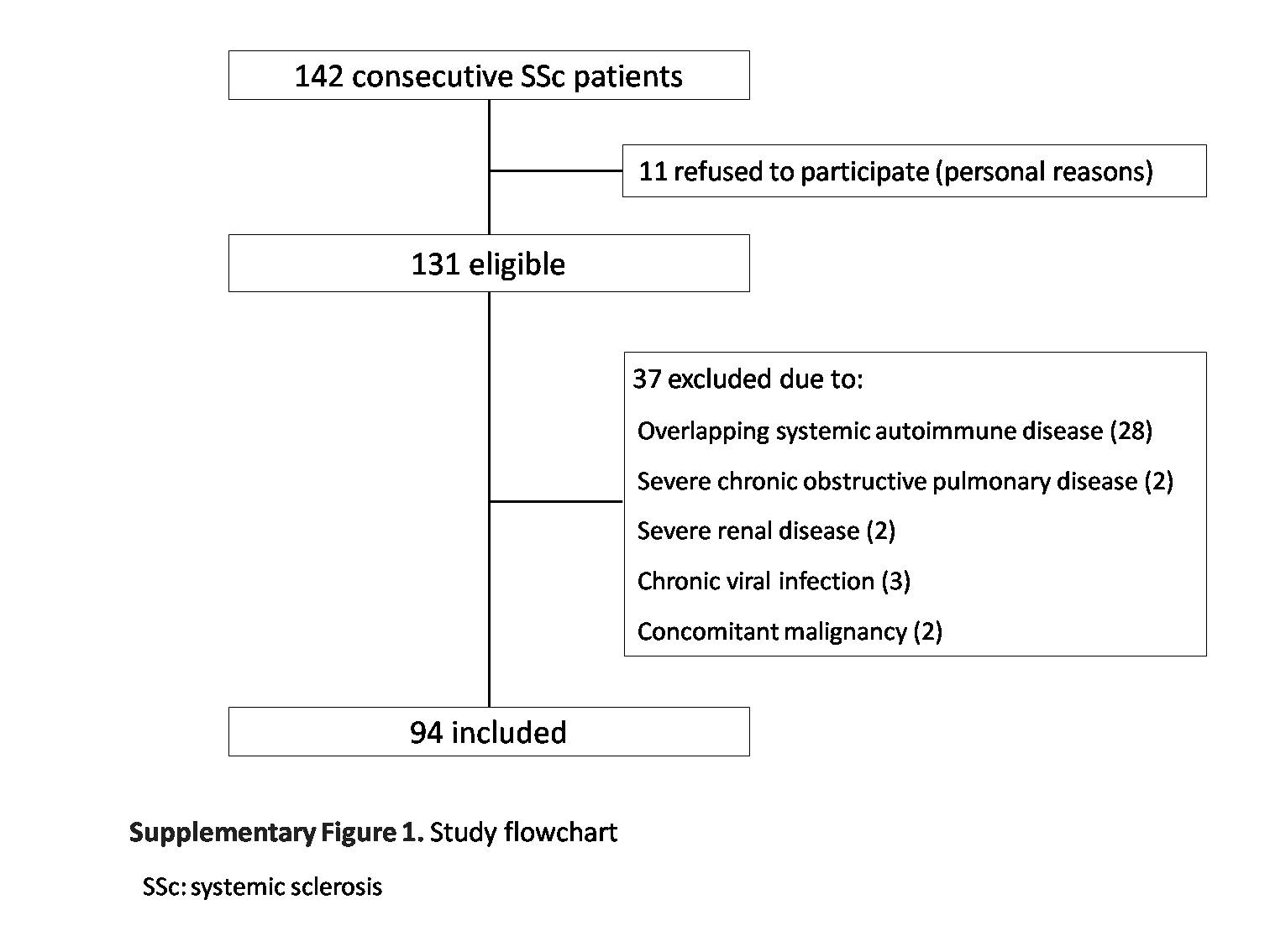

Supplement: S1 Fig — (TIF) [file pone.0245683.s002.tif]
