# Supplementary material for: Practical screening tools for sarcopenia in patients with systemic sclerosis
Source: PLoS One. 2021 Jan 22;16(1):e0245683. doi: 10.1371/journal.pone.0245683 (PMC7822499; doi:10.1371/journal.pone.0245683)
Supplement: Supplementary file 3 [file pone.0245683.s001.odt]

SARC-F – Youden Index:

A SARC-F score equal to or greater than 4 performed better in our population than a cut-off of 6: Youden index 0.21 vs. 0.10, and AUC 0.588 vs. 0.549, respectively

Raw data of each tool:

The raw data of each tool vs. the gold standard diagnostic results were provided in 2x2 tables.

SARC-F ≥ 4

|  | Presence of sarcopenia | Absence of sarcopenia | Total |
| --- | --- | --- | --- |
| SARC-F ≥ 4 | 6 | 15 | 21 |
| SARC-F < 4 | 9 | 64 | 73 |
| Total | 15 | 79 | 94 |

SARC-F ≥ 6

|  | Presence of sarcopenia | Absence of sarcopenia | Total |
| --- | --- | --- | --- |
| SARC-F ≥ 6 | 12 | 71 | 83 |
| SARC-F < 6 | 3 | 8 | 11 |
| Total | 15 | 79 | 94 |

Sensitivity			80.00% (51.91% to 95.67%)

Specificity			10.13% (4.47% to 18.98%)

Positive Likelihood Ratio		0.89 (0.68 to 1.16)

Negative Likelihood Ratio	1.97 (0.59 to 6.60)

Disease prevalence		15.96% (9.22% to 24.95%)

Positive Predictive Value		14.46% (11.49% to 18.03%)

Negative Predictive Value 	72.73% (44.38% to 89.91%)

AUC 				0.549 (0.383-0.716)

SARC-CalF

|  | Presence of sarcopenia | Absence of sarcopenia | Total |
| --- | --- | --- | --- |
| SARC-CalF ≥ 11 | 8 | 12 | 20 |
| SARC-CalF < 11 | 7 | 67 | 74 |
| Total | 15 | 79 | 94 |

SARC-F+EBM

|  | Presence of sarcopenia | Absence of sarcopenia | Total |
| --- | --- | --- | --- |
| SARC-F+EBM ≥ 12 | 9 | 11 | 20 |
| SARC-F+EBM < 12 | 6 | 68 | 74 |
| Total | 15 | 79 | 94 |

Ishii screening test

|  | Presence of sarcopenia | Absence of sarcopenia | Total |
| --- | --- | --- | --- |
| Ishii ≥105 in men and ≥120 in women | 13 | 21 | 34 |
| Ishii <105 in men and <120 in women | 2 | 58 | 60 |
| Total | 15 | 79 | 94 |
